# Clinical prognostic models for severe dengue: a systematic review protocol

**DOI:** 10.12688/wellcomeopenres.15033.2

**Published:** 2019-08-02

**Authors:** Thang Dao Phuoc, Long Khuong Quynh, Linh Vien Dang Khanh, Thinh Ong Phuc, Hieu Le Sy, Tu Le Ngoc, Lam Phung Khanh

**Affiliations:** 1 University of Medicine and Pharmacy, Ho Chi Minh City, 700000, Vietnam; 2 Oxford University Clinical Research Unit, Ho Chi Minh City, Vietnam

**Keywords:** decision support techniques, dengue, dengue hemorrhagic fever, dengue shock syndrome, severe dengue, model, forecasting, prediction, prognosis

## Abstract

**Background: **Dengue is a common mosquito-borne, with high morbidity rates recorded in the annual. Dengue contributes to a major disease burden in many tropical countries. This demonstrates the urgent need in developing effective approaches to identify severe cases early. For this purpose, many multivariable prognostic models using multiple prognostic variables were developed to predict the risk of progression to severe outcomes. The aim of the planned systematic review is to identify and describe the existing clinical multivariable prognostic models for severe dengue as well as examine the possibility of combining them. These findings will suggest directions for further research of this field.

**Methods:** This protocol has followed the guidelines of the Preferred Reporting Items for Systematic Reviews and Meta – Analyses Protocol (PRISMA-P). We will conduct a comprehensive search of Pubmed, Embase, and Web of Science. Eligibility criteria include being published in peer-review journals, focusing on human subjects and developing the multivariable prognostic model for severe dengue, without any restriction on language, location and period of publication, and study design. The reference list will be captured and removed from duplications. We will use the Critical Appraisal and Data Extraction for Systematic Reviews of Prediction Modelling Studies (CHARMS) checklist to extract data and Prediction study risk of bias assessment tool (PROBAST) to assess the study quality.

**Discussion: **This systematic review will describe the existing prediction models, summarize the current status of prognostic research on dengue, and report the possibility to combine the models to optimize the power of each paradigm.

**PROSPERO registration**: CRD42018102907

## List of abbreviations

**CHARM:** the CHecklist for critical Appraisal and data extraction for systematic Reviews of prediction Modelling Studies

**DF:** Dengue Fever

**DHF:** Dengue Hemorrhagic Fever

**DSS:** Dengue Shock Syndrome

**PRIMA:** the Preferred Reporting Items for Systematic Reviews and Meta – Analyses

**PRISMA-P:** the Preferred Reporting Items for Systematic Reviews and Meta – Analyses Protocol

**WHO:** World Health Organization

## Introduction

Dengue is the most common mosquito-borne viral infection globally and a considerable public health burden in many tropical countries ^1^. Globally, the number of dengue infection has increased rapidly within the past two decades, and it is estimated that the incidence of dengue went up six-fold from 1990 to 2013 ^2^. The disease has a wide-spectrum of clinical manifestations, from self-limited mild illness to severe dengue ^3^. Although only a small proportion of dengue-infected patients develop severe dengue, it is responsible for an average of over 9,000 deaths per year over the same time period, globally ^2^. The current WHO’s guidelines suggests 2 levels of severity: dengue with/without warning signs and severe dengue ^4^. While the former is usually a mild illness with considerable morbidity but low mortality, the latter is associated with uncompensated plasma leakage which may lead to hypovolemic shock, organ dysfunction and several complications including death ^3^.

Disease management is mainly based on early supportive treatment, since there is no specific antiviral drugs available ^3, 5, 6^. Therefore, identifying early patients who are at risk of developing severe outcomes based on simple and readily available risk factors plays an important role in dengue case management. Potential predictors could be ranged from clinical features and standard laboratory tests, including hematocrit and platelet count to viral and immunological markers as well as novel markers of endothelial activation and vascular functions ^7^. To accurately evaluate the risks of a specific clinical outcome for individual patients, a single predictor is hardly adequate, hence, there is a need to develop a multivariable prognostic model using multiple prognostic variables ^8^. Building a prediction model, which comprises three typical elements including suggested predictors, an outcome and a statistical model, is the third step of a 4-step recommended design for prognostic research ^9^.

Recently, many clinical prognostic studies for severe dengue have been published. However, there remains no widely accepted paradigm to predict disease severity. This may be due to the heterogeneity in the study populations and design, clinical definitions of “severe” dengue, candidate predictors and analytical approaches between studies. It is, therefore, necessary to conduct this systematic review to provide a general and detailed description of the status quo of clinical prediction models for severe dengue, and to suggest further development in this emerging field of research.

### Research aims

The primary aim of this systematic review is to identify and evaluate currently available multivariable prognostic models for severe dengue. To the authors’ knowledge, this is the first time that multivariable prognostic models for severe dengue have been reviewed systematically. The secondary purpose is to establish if there is any possibility to combine these models. Furthermore, some suggestions will be given on how to develop robust and applicable clinical prediction models for severe dengue.

## Methods

This protocol has been prepared using the Preferred Reporting Items for Systematic Reviews and Meta – Analyses Protocol (PRISMA-P) ^10^. The PRISMA-P checklist is available as part of the Reporting guidelines ^11^.

### Selection criteria

***Study design.*** We will include all published articles on developing, validating or updating the multivariable prognostic models aiming to predict the risk of death or severe dengue based on the WHO 1997 and 2009 dengue case classification. There is no restriction on language, time of publication, country in which the study was conducted or study setting. Book chapters, commentaries, conference abstract, reviews, editorials, guidelines and letters will be excluded.

***Population.*** This review will include studies published in peer-reviewed journals and conducted on patients who were diagnosed with dengue infection based on clinical features and/or dengue diagnostic tests such as but not limited to detection of antigen (NS1), serological test (total IgM, IgG ELISA), nucleic acid detection (RT-PCR, real-time RT-PCR) and virus isolation with the aim to develop, validate or update the multivariable prognostic models to predict the risk of occurrence of severe dengue. “Severe dengue” term for this study entails: Dengue Hemorrhagic Fever (DHF), Dengue Shock Syndrome (DSS) of the 1997 WHO dengue case classification and Severe Dengue of 2009 WHO dengue case classification, and mortality.

***Types of multivariable prognostic models.*** Each included studies must be conducted to develop, validate or update a clinical multivariable prognostic model in order to predict the risk of occurrence of severe dengue for patients who have any symptoms relating to dengue infection or diagnosed with dengue infection. The developed model must involve at least two predictors. We will take into consideration all types of prognostic model development studies (e.g. with or without external validation in independent data, and with or without model updating).

### Primary and secondary outcomes

The primary outcomes of this systematic review will be the number of currently available clinical multivariable prognostic models for severe dengue and their properties including: study designs, outcomes, candidate predictors, statistical approach, validation status, and model performance. The secondary outcome of this systematic review is the possibility to combine these identified models into a more comprehensive prediction model.

### Search strategy

We will conduct a comprehensive search to identify all related publications. The following bibliographic databases will be searched by individual search strategies created specifically for each database: PubMed, Embase, and Web of Science. PubMed, developed by the US National Library of Medicine, National Institutes of Health, covers the majority of biomedical journals published from 1950, and unlimited keywords allowed ^12^. Embase and Web of Science also includes diverse published journals ^13^, with languages and keyword supported search. While Embase covers extensive Europe journals, Web of Science provides articles published from 1900, and citation analysis ^12^.

Since the patient characteristics are broad, to build up the search strategy, we will focus our keywords on two main parts: (1) “prognostic model” for clinical multivariable prognostic model, and (2) “severe dengue” for outcome relating to DHF, DSS of the WHO 1997, severe dengue of the WHO 2009 dengue case classification and mortality. The search strategies will be designed by combining index terms and text words relating to two main parts mentioned. All possible synonyms of these terms will be identified and included to cover more comprehensively the review subject. We will use Boolean operator “OR” to link all index terms, text words, and synonyms into particular groups relating to main keywords and Boolean operator “AND” to link all groups into the final search string. Search fields will be applied to make the search string more appropriate to each database. We will only focus on the articles relating to human subjects. The Boolean operator “NOT” will be also used to exclude book chapters, documents, editorials, review and guidelines. There is no restriction on language and publication date. The sample search strategy developed for PubMed, Embase and Web of Science as Extended data ^11^.

### Selection of studies

Study selection will be conducted by following the PRISMA flow diagram. We will capture the reference lists from each database and then import them into Mendeley software to remove duplications. The BibTex file containing all filtered references will be then exported. We will import the BibTex file into JabRef software to create the Excel file as the reference list. The recorded data will be managed by the reference lists throughout the review process. Two independent reviewers will screen simultaneously all titles and abstracts based on the following broad screening criteria: (1) focusing on human subjects, (2) focusing on developing the clinical prognostic model, (3) focusing on severe dengue outcome (DHF, DSS of the 1997 WHO dengue case classification and Severe Dengue of 2009 WHO dengue case classification, and mortality). We will exclude articles that do not meet the screening criteria above. Any discrepancies reported during the study screening will be resolved through discussion with a third reviewer. Full-texts of identified abstracts will be retrieved and reviewed to check for more detailed criteria: (1) focusing on developing a multivariable model relating to severe dengue, (2) focusing on patients who were diagnosed with dengue infection based on clinical features and/or dengue diagnostic test. Identified publication will be excluded if full-text is not available. If any disagreements occur, we will reach a consensus by consulting a third reviewer. The reasons for excluding the articles will be reported in detail. Attempts will be made to translate the non-English articles into English.

### Data extraction

Extracting data will be performed by two independent reviewers to collect essential data from full texts. The form for extracting data will be designed based on the Checklist for Critical Appraisal and Data Extraction for Systematic Reviews of Prediction Modelling Studies (CHARMS). The CHARMS checklist was developed by Moons *et al*. to provide guidance on framing of systematic data extraction forms with eleven specific domains ^14^. Additional data on article information, source of data, candidate predictors, sample size and model characteristics will also be extracted as suggested in the Cochrane Methods Prognosis Template ^15^. All data retrieved from identified full-texts will be imported into the in-depth form. Any discrepancy will be resolved by discussing with the third reviewer to reach a consensus.

We will extract the following data as a minimum:

- Article information: author, year of publication, country of publication, location
- Source of data: study design (e.g. prospective or retrospective, case – control, etc) , follow-up time, study context (in primary or secondary health care center)
- Participants: patient characteristics, for example, age, gender, dengue diagnosis criteria, comorbidity, given treatment, etc. recruitment method, number of study centers, patient inclusion, and exclusion criteria.
- Outcome(s) to be predicted: severe dengue definition, for example, DHF, DSS, and severe dengue of 1997 and 2009 WHO dengue case classification, mortality, shock recurrence; method for outcome measurement, time of outcome occurrence
- Candidate predictors: number and type of predictors, definition and collection method of predictors, timing of measurement, measures of association and predictive performance (e.g. risk ratio, odds ratio, hazard ratio or mean difference), handling of predictor in model
- Sample size: total number of participants, ratio of participants to candidate predictors,
- Missing data: number and percentage of missing value in total and for each predictor, extend of loss to follow-up, method of dealing with missing data (e.g. imputation)
- Model development: modelling method (e.g. statistical model, machine learning techniques), variable selection method (e.g. best subset, stepwise selection), selection criteria (e.g. P-value, AIC, BIC)
- Model performance: calibration (e.g. calibration slope), discrimination (e.g. AUC, C-statistic, D-statistic) and classification measures (accuracy, sensitivity, specificity, predictive values)
- Model evaluation: internal and external validation
- Model presentation: how the final model was presented (e.g. nomogram, score chart)
- Model applicability and interpretation: strengths, limitations and applicability in clinical setting

### Assessment of study quality

All identified studies meeting the eligibility criteria will be assessed based on the domains suggested by Prediction study Risk Of Bias Assessment Tool (PROBAST), the currently recommended tool for assessing the risk of bias in prognostic studies ^16^. This tool was built up by experts in the field on the basis of the Delphi process comprising five domains: participant selection, predictors, outcome, sample size, and participant flow and analysis, with detailed guidance for determining potential items that could possibly be biased ^16^. The quality of identified studies will be evaluated by two independent reviewers. Discrepancies will be resolved by discussing with the third reviewer.

### Data synthesis

We will descriptively analyze all clinical multivariable prognostic models by describing and narratively synthesizing the article and model information. No formal meta – analysis will be conducted to summarize the model performance across identified articles. The percentage and frequency of each item will be summarized and tabulated. Each model will be narratively synthesized in terms of sample size, candidate predictors, development method, performance and evaluation, presentation, applicability in clinical practice and risk of bias. From the retrieved information, we will describe the clinical heterogeneities in study participants, candidate predictors, outcomes as well as the methodological heterogeneities in the sources of data, missing data, and statistical approaches. If data on measures of association and predictive performance is insufficient, we will estimate this data using the approaches suggested by Cochrane Collaboration and Parmar *et al*. ^17, 18^. We will also explore the possibility of combining identified prediction models into a more comprehensive model using a recent meta-analysis approach ^19^. The trend in the development of prognostic models for severe dengue from the identified publications throughout years will be reported to make suggestions for further research in this field.

### Dissemination of information

Our research findings will be presented at scientific conferences and published in peer-reviewed scientific journals. We will also disseminate the findings on popular science newspapers.

### Study status

The publication search has been completed. Currently, we are conducting screening identified publications.

## Discussion

The early and proper identification of patients who are at risk of severe dengue requires processing inputs from a variety of clinical signs, examination results, and epidemiological characteristics. While clinicians may find it challenging and overwhelming to utilize such a volume of information, prediction models can help as handy tools to support in monitoring, decision making and treating dengue patients. Given the emerging prediction models in dengue infection and heterogeneous characteristics of models and algorithms, we hope this review will provide a systematic evaluation of the existing literature in the field. From our review, recommendations could be made on how to develop and report prediction models for severe dengue, how to use these models and where to focus for research in the field in near future.

## Declarations

### Data availability

***Underlying data.*** No data is associated with this article.

***Extended data.*** We have submitted our extended data into Havard Dataverse

Harvard Dataverse: Extended data “Clinical prognostic models for severe dengue: a systematic review protocol”; https://doi.org/10.7910/DVN/MU4QLJ ^11^.

Licence: CC0 1.0 Universal (CC0 1.0) Public Domain Dedication

### Reporting guidelines

Harvard Dataverse: PRISMA-P checklist for “Clinical prognostic models for severe dengue: a systematic review protocol” https://doi.org/10.7910/DVN/MU4QLJ ^11^.

Licence: CC0 1.0 Universal (CC0 1.0) Public Domain Dedication

## References

[1] Nathan MB Dayal-Drager R Guzman M: Epidemiology, burden of disease and transmission. WHO *Dengue guidelines for diagnosis, treatment, prevention and control* New edition Geneva: WHO.2009;1–21. Reference Source

[2] Stanaway JD Shepard DS Undurraga EA: The global burden of dengue: an analysis from the Global Burden of Disease Study 2013. *Lancet Infect Dis.* 2016;16(6):712–23. 10.1016/S1473-3099(16)00026-8 26874619 PMC5012511

[3] Simmons CP Farrar JJ Nguyen vV: Dengue. *N Engl J Med.* 2012;366(15):1423–32. 10.1056/NEJMra1110265 22494122

[4] World Health Organization: Dengue Guidelines for Diagnosis, Treatment, Prevention and Control. 2009 ^th^edition. France: World Health Organization;2009 Reference Source 23762963

[5] Whitehorn J Yacoub S Anders KL: Dengue therapeutics, chemoprophylaxis, and allied tools: state of the art and future directions. *PLoS Negl Trop Dis.* 2014;8(8):e3025. 10.1371/journal.pntd.0003025 25166493 PMC4148227

[6] World Health Organization: Handbook for clinical management of dengue. Report No ISBN. 2015; 978.2012 Reference Source

[7] Yacoub S Wills B: Predicting outcome from dengue. *BMC Med.* 2014;12:147. 10.1186/s12916-014-0147-9 25259615 PMC4154521

[8] Moons KG Royston P Vergouwe Y: Prognosis and prognostic research: what, why, and how? *BMJ.* 2009;338:b375. 10.1136/bmj.b375 19237405

[9] Hemingway H Croft P Perel P: Prognosis research strategy (PROGRESS) 1: a framework for researching clinical outcomes. *BMJ.* 2013;346:e5595. 10.1136/bmj.e5595 23386360 PMC3565687

[10] Moher D Shamseer L Clarke M: Preferred reporting items for systematic review and meta-analysis protocols (PRISMA-P) 2015 statement. *Syst Rev.* 2015;4:1. 10.1186/2046-4053-4-1 25554246 PMC4320440

[11] Thang DP: Clinical prognostic models for severe dengue: a systematic review protocol. *Harvard Dataverse, V2.* 2019 10.7910/DVN/MU4QLJ PMC6694715 31448337

[12] Falagas ME Pitsouni EI Malietzis GA: Comparison of PubMed, Scopus, Web of Science, and Google Scholar: strengths and weaknesses. *FASEB J.* 2008;22(2):338–42. 10.1096/fj.07-9492LSF 17884971

[13] Wong SS Wilczynski NL Haynes RB: Developing optimal search strategies for detecting clinically sound treatment studies in EMBASE. *J Med Libr Assoc.* 2006;94(1):41–7. 16404468 PMC1324770

[14] Moons KG de Groot JA Bouwmeester W: Critical appraisal and data extraction for systematic reviews of prediction modelling studies: the CHARMS checklist. *PLoS Med.* 2014;11(10):e1001744. 10.1371/journal.pmed.1001744 25314315 PMC4196729

[15] Review Tool. Reference Source

[16] Wolff RF Moons KGM Riley RD: PROBAST: A Tool to Assess the Risk of Bias and Applicability of Prediction Model Studies. *Ann Intern Med.* 2019;170(1):51–8. 10.7326/M18-1376 30596875

[17] Parmar MK Torri V Stewart L: Extracting summary statistics to perform meta-analyses of the published literature for survival endpoints. *Stat Med.* 1998;17(24):2815–34. 10.1002/(SICI)1097-0258(19981230)17:24<2815::AID-SIM110>t;3.0.CO;2-8 9921604

[18] The Cochrane Collaboration: Cochrane handbook for systematic reviews of interventions. *Cochrane handbook for systematic reviews of interventions.* 2008 Reference Source

[19] Debray TP Koffijberg H Nieboer D: Meta-analysis and aggregation of multiple published prediction models. *Stat Med.* 2014;33(14):2341–62. 10.1002/sim.6080 24752993

