# Supplementary material for: Clinical prognostic models for severe dengue: a systematic review protocol
Source: Wellcome Open Res. 2019 Aug 2;4:12. Originally published 2019 Jan 24. [Version 2] doi: 10.12688/wellcomeopenres.15033.2 (PMC6694715; doi:10.12688/wellcomeopenres.15033.2)
Supplement: Peer review history

# Peer review: Reviewer response for version 2

**DOI:** 10.21956/wellcomeopenres.16820.r36128

**Published:** 2019-08-20

**Authors:** Olaf Horstick

**Affiliations:** 1 Heidelberg Instititue of Global Health, University of Heidelberg, Heidelberg, Germany

Good now and good luck with the research.

I confirm that I have read this submission and believe that I have an appropriate level of expertise to confirm that it is of an acceptable scientific standard.

# Peer review: Reviewer response for version 2

**DOI:** 10.21956/wellcomeopenres.16820.r36129

**Published:** 2019-08-09

**Authors:** Bushra Khalid

**Affiliations:** 1 Evolution and Ecology Program, International Institute for Applied Systems Analysis, Laxenburg, Austria

I would recommend the protocol for indexing.

I confirm that I have read this submission and believe that I have an appropriate level of expertise to confirm that it is of an acceptable scientific standard.

# Peer review: Reviewer response for version 1

**DOI:** 10.21956/wellcomeopenres.16401.r34670

**Published:** 2019-02-14

**Authors:** Olaf Horstick

**Affiliations:** 1 Heidelberg Instititue of Global Health, University of Heidelberg, Heidelberg, Germany

This is a very good protocol for the systematic review. I would only suggest to include searches on google scholar, with a modified search strategy on this database, and also searches of grey literature. The topic may well be in national guidelines, etc. and not in published scientific literature only.

I confirm that I have read this submission and believe that I have an appropriate level of expertise to confirm that it is of an acceptable scientific standard.

# Response

**Published:** 2019-07-30

**Authors:** Thang Dao Phuoc

I thank you for your helpful comments. Your suggestions are great and worth considering. However, we currently do not have enough time to search for more databases as well as grey literature.

# Peer review: Reviewer response for version 1

**DOI:** 10.21956/wellcomeopenres.16401.r34669

**Published:** 2019-02-12

**Authors:** Bushra Khalid

**Affiliations:** 1 Evolution and Ecology Program, International Institute for Applied Systems Analysis, Laxenburg, Austria

This is a study protocol for identifying the clinical prognostic models for dengue management. Authors intend to identify already developed prognostic models for dengue management and check the possibility of combining them in order to increase the efficiency in predictability for an early treatment. This is an interesting study design especially from the perspective of combining several prognostic models however the first part of the study requires more description to fully understand what authors want to carry out in this work.

1. There are several grammatical and sentence structure errors in the document.
2. “The number of dengue infection has increased…” this sentence in the introduction does not state the region (any particular continent/global/particular region) on increase of dengue infection. The period 1990-2013 for citing in the current research is quite old. New research may be cited from the scientific literature.
3. “it is responsible for an average of over 9,000 deaths per year….” this sentence does not state the region of 9,000 deaths per year.
4. After mentioning the rate of dengue, the severity of dengue has been stated all of a sudden. It requires mentioning more information beforehand regarding the severity of dengue fever. Authors should consider maintaining a rhythm of the topic of concern.
5. Introduction section should be revised considerably to inform the background of the intended study.
6. The objective of the study does not clearly mention whether the study intends to identify the severe dengue within an individual patient or is it a collective term for a severe dengue incidence within a region?
7. Authors should describe the term “multivariate prognostic”.
8. Authors should revise the methods section and eliminate repetition in the text.
9. Authors may consider introducing any such multivariate prognostic models, if they exist.
10. Authors may consider discussing a little bit of prognostic variables and their importance in the transmission of severe dengue to highlight the importance of conducting this study.
11. As authors stated that “there remains no widely accepted paradigm to predict disease severity…” how do authors defend carrying out this study based on the various global disease predictive models which are not well-designed/functional? Why and how this study would be useful and applicable in reality for policy making or treatment.
12. Various prognostic models may have used various variables, how do authors think to compare the different situational/regional/global models with each other since every region/situation cannot be comparable.

I confirm that I have read this submission and believe that I have an appropriate level of expertise to confirm that it is of an acceptable scientific standard, however I have significant reservations, as outlined above.

# Response

**Published:** 2019-07-30

**Authors:** Thang Dao Phuoc

I thank you for your helpful comments. We have edited some details based on your suggestions and have some of the following responses to your suggestions.

**1. There are several grammatical and sentence structure errors in the document.**

We have checked again our protocol and identified some grammatical errors and sentence structure errors. We have already fixed it in our revision.

**2. “The number of dengue infection has increased…” this sentence in the introduction does not state** **the region (any particular continent/global/particular region) on increase of dengue infection. The** **period 1990-2013 for citing in the current research is quite old. New research may be cited from the** **scientific literature.**

This is a global trend, from data of 76 countries and 1636 case reports of dengue, following the cited reference. To clarify that, we have modified the sentence to: “Globally, the number of dengue infection has increased …”. Even though the period 1990-2013 seems quite old, this research was published in 2016 and according to our knowledge, this is the standard and most up-to-date reference on this topic.

**3. “it is responsible for an average of over 9,000 deaths per year….” this sentence does not state the** **region of 9,000 deaths per year.**

Similar to question number 2, the number of deaths per year was estimated throughout data of 130 countries over the world. To clarify that, we have modified the sentence to: “it is responsible for an average of over 9,000 deaths per year over the same time period, globally.”

**4. After mentioning the rate of dengue, the severity of dengue has been stated all of a sudden. It** **requires mentioning more information beforehand regarding the severity of dengue fever. Authors** **should consider maintaining a rhythm of the topic of concern.**

We have added this sentence to briefly mention about dengue severity after the sentence about global rate of dengue:

“"The disease has a wide-spectrum of clinical manifestations, from self-limited mild illness to severe dengue [reference: Simmons *et al.* (2012)]"”

**5. Introduction section should be revised considerably to inform the background of the intended** **study.**

As presented in our protocol, the introduction is divided into 3 main parts, namely:

1/ Global epidemiology of dengue – Paragraph 1: line 1 – line 14

2/ Difficulties of dengue management and the importance in early predicting severe dengue progression – Paragraph 2: line 15 – line 30

3/ Limitations of clinical prognostic studies – objectives of systematic review

Unless there is any specific background information you suggest that we should add, we believe that this structure and the presented introduction section provide adequate and appropriate information as background of our study.

**6. The objective of the study does not clearly mention whether the study intends to identify the severe** **dengue within an individual patient or is it a collective term for a severe dengue incidence within a** **region?**

As mentioned in our protocol, our objective is to describe in detail the status of existing clinical prediction models, mainly focusing on prognosis, for predicting risk of severe dengue progression, and to suggest possible development for this field of research. To our knowledge, “clinical prognostic model” or “clinical prediction model” are current standard terms that refer to models aiming to predict the risk of future clinical outcomes in individual patients (Reference: Steyerberg EW *et al.* (2013) Prognosis Research Strategy (PROGRESS) 3: Prognostic Model Research. Plos Med, 10(2): e1001381, URL: https://www.ncbi.nlm.nih.gov/pmc/articles/PMC3564751/.

**7. Authors should describe the term “multivariate prognostic”.**

Of note, we did not use the term “multivariate prognostic” but rather the term “multivariable prognostic models” in our manuscript (we believe that statistically, there are major differences between these two terms, “multivariable” and “multivariate”). “Multivariable prognostic models” refers to prognostic models which are multivariable, which in turn is a standard term that refers to statistical regression models with more than one predictor (independent variables) (Reference: Hidalgo B and Goodman M (2013) Multivariate or Multivariable Regression?. Am J Public Health, 103(1):39-40).

**8. Authors should revise the methods section and eliminate repetition in the text.**

We have checked the methods section multiple times for repetition. Even though there are some words/phrases look similar to each other, they are actually different and refer to different things. Therefore, we still keep this section as it currently stands.

**9. Authors may consider introducing any such multivariate prognostic models, if they exist.**

We have introduced a recent such model in the first sentence of the fourth paragraph in the introduction. The sentence now reads as “Recently, many clinical prognostic studies for severe dengue have been published, such as the Early Severe Dengue Identifier, a simple 4-predictor algorithm to predict individual risk of severe dengue [Reference: Tuan NM *et al.* (2017) An Evidence-Based Algorithm for Early Prognosis of Severe Dengue in the Outpatient Setting. Clinical Infectious Diseases, 64(5): 656–663]”.

**10. Authors may consider discussing a little bit of prognostic variables and their importance in the** **transmission of severe dengue to highlight the importance of conducting this study.**

In the second paragraph of the manuscript, we already briefly mentioned about considered prognostic variables of severe dengue: “Potential predictors could be ranged from clinical features and standard laboratory tests, including hematocrit and platelet count to viral and immunological markers as well as novel markers of endothelial activation and vascular functions”.

Transmission of dengue plays an important role in dengue diagnosis and public health intervention; however, “transmission of severe dengue” seems not to be very relevant to our study. Even though there is some evidence to suggest that specific dengue serotype (especially serotype DENV-2) is more associated with severe dengue than other serotypes [Simmons C *et al.* (2012) Dengue. NEJM, 366(15):1423-32], severe dengue is rather a consequence of a complex interaction between viral and host factors than a separated entity which can be transmitted.

**11. As authors stated that “there remains no widely accepted paradigm to predict disease severity…”** **how do authors defend carrying out this study based on the various global disease predictive** **models which are not well-designed/functional? Why and how this study would be useful and** **applicable in reality for policy making or treatment.**

The fact that “there remains no widely accepted paradigm to predict disease …” does not mean that current predictive models in the field are “not well-designed/functional”. We believe that this fact generally means two things: (1) the topic is still an open field for research and exploration, and (2) a high heterogeneity in how research is conducted and reported is expected. High heterogeneity in research design and report makes it extremely difficult to aggregate research findings in order to draw overall conclusions or develop a care management policy that is applicable. For this reason, we conduct this systematic review to identify this heterogeneity by detailed describing of published research and carefully assess their quality. Our findings would then be the basis for further attempts to harmonize and standardize how prognostic models for severe dengue are developed and reported. Together with other similar movements (such as the CONSORT statement for reporting randomized controlled trials), this piece of research would be one of the important factors to maintain and improve the quality of research as well as facilitate research progress in this field.

**12. Various prognostic models may have used various variables, how do authors think to compare the different situational/regional/global models with each other since every region/situation cannot be comparable**

Heterogeneity in included predictors is a big issue for any attempt to compare or combine different prognostic models. In terms of comparison, we acknowledge this heterogeneity and thus focus more on comparing study designs, methods for model development and model presentation. In terms of combination, we will first explore the possibility to combine published models using approaches described in Debray *et al.* (2014) Meta‐analysis and aggregation of multiple published prediction models. Statistics in Medicine, 33(14):2341-62, namely “model averaging” and “staked regressions”, which allow combining models with different sets of predictors.
